# A facile method for anti-cancer drug encapsulation into polymersomes with a core-satellite structure

**DOI:** 10.1080/10717544.2022.2103209

**Published:** 2022-07-29

**Authors:** Hongchao Xu, Weiwei Cui, Zhitao Zong, Yinqiu Tan, Congjun Xu, Jiahui Cao, Ting Lai, Qi Tang, Zhongjuan Wang, Xiaofeng Sui, Cuifeng Wang

**Affiliations:** a Department of Neurosurgery, Shenzhen Hospital and The Third School of Clinical Medicine, Southern Medical University, Shenzhen, Guangdong, P. R. China; b School of Pharmaceutical Sciences, Sun Yat-sen University, Guangzhou, P. R. China; c Department of neurosurgery, JiuJiang Hospital of Traditional Chinese Medicine, Jiujiang, P. R. China; d Department of Pharmacy, Yan’an Hospital Affiliated to Kunming Medical University, Kunming, P. R. China; e College of Chemistry, Chemical Engineering and Biotechnology, Donghua University, Shanghai, P. R. China

**Keywords:** Polymersome, direct hydration method, core-satellite drug encapsulation, glioma, multidrug resistance

## Abstract

Polymersomes possess the self-assembly vesicular structure similar to liposomes. Although a variety of comparisons between polymersomes and liposomes in the aspects of physical properties, preparation and applications have been elaborated in many studies, few focus on their differences in drug encapsulation, delivery and release *in vitro* and *in vivo.* In the present work, we have provided a modified direct hydration method to encapsulate anti-cancer drug paclitaxel (PTX) into PEG-*b*-PCL constituted polymersomes (PTX@PS). In addition to advantages including narrow particle size distribution, high colloid stability and moderate drug-loading efficiency, we find that the loaded drug aggregate in small clusters and reside through the polymersome membrane, representing a unique core-satellite structure which might facilitate the sustained drug release. Compared with commercial liposomal PTX formulation (Lipusu^®^), PTX@PS exhibited superb tumor cell killing ability underlain by multiple pro-apoptotic mechanisms. Moreover, endocytic process of PTX@PS significantly inhibits drug transporter P-gp expression which could be largely activated by free drug diffusion. In glioma mice models, it has also confirmed that PTX@PS remarkably eradicate tumors, which renders polymersomes as a promising alternative to liposomes as drug carriers in clinic.

## 1 Introduction

Nanosized drug delivery systems have attracted increasing research interests in the past decades. More and more nano-formulations are being approved for marketing or clinical use, since they display superb drug efficacy or therapeutic effect compared with the standard formulations (Patra et al., 2018). Among various nano-formulations, liposomes are one of the most extensively investigated drug carriers for improving the delivery of therapeutic agents in diverse diseases (Large et al., 2021). The structure of liposomes consists of a hydrophilic interior and an amphiphilic lipids bilayer, therefore it allows hydrophobic compounds inserting into the hydrophobic bilayer, and hydrophilic compounds encapsulated in the larger interior. Although lipid formulations on the market have shown the enormous therapeutic benefits to deliver drug and gene, such as Doxil^®^, Exparel^®^, ONPATTRO^®^, some drawbacks of liposomes in manufacture and development are still needed to overcome, including stability issues, low drug entrapment, particle size control, batch to batch reproducibility and short circulation time (Laouini et al., 2012; Thi et al., 2021), which opens a new avenue for investigation of advanced drug delivery systems.

Polymersomes, similar to liposomes structure, possess a large watery interior surrounding with a tough bilayer membrane assembled from amphiphilic block polymers, thereby they are also versatile for encapsulation and delivery of both hydrophilic (e.g. proteins, siRNA, DNA) and hydrophobic drug (e.g. paclitaxel, doxorubicin) (Lee & Feijen, 2012; Peters et al., 2014; Rideau et al., 2018). In addition to advantages common to most liposomes-based drug delivery systems, polymersomes displayed many unique features to improve the drug encapsulation and stability over liposomes (LoPresti et al., 2009). Compared with small phospholipid molecules, higher molecular weights polymers are able to form a thicker membrane (in the range of 5–30 nm versus 3–5 nm for liposomes) (Le Meins et al., 2011), which will enhance the mechanical strength and impermeability, therefore, it could not increase the loading capacity of hydrophobic drug, but also decrease the drug leakage from the hydrophobic layer of the membranes (Matoori & Leroux, 2020). Moreover, the tailored polymers are able to control the rate of cargo release by tuning the molecular weights and hydrophobicity. Thicker membranes may result in slower release rates of hydrophobic substrates probably due to higher diffusional distances. Collectively, polymersomes show a superior stability and flexibility which renders them as a suitable candidate for replacing liposomes.

Polymersome preparation is also similar to that of liposomes, usually using solvent displacement method and thin film hydration method (Chidanguro et al., 2018). In general, the polymer is dissolved in an organic solvent to form an organic phase that is added to an aqueous solution (aqueous phase) by the mixing step, which enables a fine dispersion of the polymer in the aqueous phase and the subsequent self-assemble of polymersomes. Except of these methods with disadvantages in terms of time, cost and facilities, the polymersomes are produced at very low concentrations but contaminated with high amounts of residual solvent leading to great challenges to scalability and translation. In our previous work (Sui et al., 2015), we have developed a modified direct hydration method to prepare the PEG-*b*-PCL polymersomes under mild conditions (50 °C, ∼ 1 h) without any organic solvent, sonication or freeze-thawing steps involved. Therefore, the yielded polymersomes with high quality and well-controlled size are highly suitable for the delivery of both insoluble drug and bioactivate macromolecules. Although previous studies (Ridolfo et al., 2018; Cao et al., 2021) have applied direct hydration method to encapsulate drug, the obtained nanoformulations were micellar rather polymersomal structure. Additionally, it has not been investigated yet that encapsulation performance and release profile of polymersomes compared with liposomes to evaluate their therapeutic potential as drug carriers.

Due to its poor solubility, anti-cancer drug Paclitaxel (PTX) is either formulated in injection (Taxol^®^) using the excipient Cremaphor EL associated with toxic effects (Utreja et al., 2012), or developing nanocarriers such as albumin-entrapped PTX (Abraxane™), polymeric micelles (NK105 and Genexol-PM™) and liposomal formulations (Lipusu^®^). These nanoformulations exert only moderate therapeutic effects but hardly replace Taxol^®^. In the current study, we employ Paclitaxel (PTX) as a model drug encapsulated into polymersomes via direct hydration method, and the obtained PTX-loaded polymersomes (PTX@PS) show the considerable drug loading capacity and sustained drug release. Moreover, compared with commercial PTX liposomal formulations Lipusu^®^ as named PTX@LS in this study, PTX@PS showed higher stability, and significant in vitro and in vivo anticancer effects. Hence, this rapid and efficient method holds great potential to scale up the production of polymersomes for drug delivery.

## 2. Materials and methods

### 2.1. Materials

ε-Caprolactone (ε-CL, 99%, Sigma-Aldrich) was stirred over CaH_2_ for 24 h at room temperature and then it was distilled at reduced pressure under nitrogen. α-Methoxy-poly(ethylene glycol)_44_-ω-hydroxide (MeO–PEG_44_–OH, Mn = 2000 g/mol) was dried by co-evaporation with anhydrous toluene using a rotary evaporator. Methane sulfonic acid (MSA) was purchased from Sigma-Aldrich, and was used without further purification. Rabbit α-tubulin antibody and mouse β-Actin were purchased from Sigma-Aldrich; rabbit anti-CD31 (GB11063-2) was purchased from Servicebio; rabbit anti-Bcl-2 antibody and rabbit anti-Ki67 were purchased from Bioss; rabbit anti-MDR1/ABCB1 (E1Y7B) antibody was purchased from Cell Signaling Technology; horseradish peroxidase (HRP)-conjugated secondary antibodies were obtained from Cell signaling Technology. LysoTracker^®^ Red DND-99 and DAPI were obtained from Thermo Scientific. Reactive oxygen species (ROS) detection kit and Annexin V-FITC apoptosis detection kit were purchased from Biovision. ECL western blotting detection kit was purchased from Millipore.

### 2.2. Synthesis of poly(ethylene glycol)-block-poly (ε-caprolactone) PEG-b-PCL

Dry MeO-PEG_44_-OH was dissolved in toluene in a Schlenk flask, and then were mixed with the desired amount of CL for further equilibration at 30 °C for 10 min. MSA (molar ratio with MeO-PEG_44_-OH) was then added into the reaction mixture and allowed to stir at 30 °C for 2.5 h. After cooling to room temperature, the mixture was treated with Amberlyst^®^ A21 in order to remove the catalyst. The resin was removed by filtration and the product was precipitated in excess cold hexane. The crude product was dissolved in THF and precipitated in excess cold hexane again twice. Molecular weight of PEG-*b-*PCL was determined by gel permeation chromatography (GPC). Proton Nuclear magnetic resonance (^1^H NMR) spectra was recorded with a Bruker Avance-400 MHz spectrometer. Chemical shifts in ^1^H NMR spectra were reported in parts per million (ppm, δ) downfield from the internal standard Me_4_Si (TMS, δ = 0 ppm). ^1^H NMR (400 MHz, CDCl_3_): δ 4.01 (*t*, *J* = 6.6 Hz, 2H), 3.59 (*s*, 4H), 2.26 (*t*, *J* = 7.5 Hz, 2H), 1.59 (ddd, *J* = 9.8, 7.3, 3.0 Hz, 4H), 1.44–1.26 (m, 2H).

### 2.3. Preparation of PTX-loaded PEG-*b*-PCL polymersomes

PEG-*b*-PCL polymersomes were prepared by direct hydration method as described previously (Sui et al., 2015). Briefly, 0.5 mg of PTX, 10 mg of PEG-*b-*PCL and 100 mg of PEG 550 were weighed into a 1.5 mL centrifuge tube, heated at 60 °C, and stirred with 300 rpm speed for 20 min. After the sample solution was cooled to 50 °C, 100 μL of MiliQ water was added and stirred for further 30 min. Then 200 and 700 μL of water were added, with mixing after each addition. The polymersome emulsion (polymer concentration: 9 mg/mL) was passed through a filter (0.22 μm) and purified with an ultracentrifuge tube (cutoff 3.5 kDa).

The preparation of PEG-*b*-PCL polymersomes by a thin-film hydration method followed a modified literature procedure (Rideau et al., 2018). In brief, 10 mg of PEG-*b*-PCL and 0.5 mg of PTX were dissolved in 6 mL chloroform and put into a round bottom flask. Then, the solvent was removed by rotary evaporation under reduced pressure to form a thin film. Any trace of residual solvent was then evaporated under nitrogen. The dried film was hydrated with 10 mL of water under sonication. The obtained polymersomes suspension was purified with an ultracentrifuge tube (cutoff 3.5 kDa).

### 2.4. Particle size and zeta potential

Particle size and zeta potential of PEG-*b*-PCL polymersomes were measured using dynamic light scattering (DLS) and electrophoretic light scattering on a Zetasizer NS90 (Malvern Instruments, UK), respectively. Three independent DLS measurements of each sample were repeated using a 4 Mw He-Ne laser at a wavelength of 633 nm and a scattering angle of 90° at 25 °C.

### 2.5. Morphology analysis

Morphological of PEG-*b*-PCL polymersomes was observed with transmission electron microscope (TEM). For the TEM imaging, 10 μL of polymersomes solution was dropped onto a copper grid and then stained with 2% Phosphomolybdic acid hydrate. Next, the grids were allowed to air dry before being observed using a JET1400 transmission electron microscope at a voltage of 120 kV.

### 2.6. Drug loading and encapsulation efficiency

PTX-loaded polymersomes (20 μL) were dissolved in 180 μL of Methanal followed by centrifugation at 3000 rpm for 20 min. The supernatant was passed through a 0.22-μm filter, and then was subjected to analyze with a UV spectrophotometer (Shimadzu UV2600, Japan). The PTX concentration was determined by measuring the absorption at λ_max_ value of 227 nm.

The percentage of loading efficiency (LE) and entrapment efficiency (EE) was calculated by using the following formulas.

$$LE\;(\%)=\frac{\text{Amount of PTX found in the polymersomes }}{\text{Amount of polymersomes}}\times 100$$

$$EE\;(\%)=\frac{\text{Amount of PTX found in the polymersomes}}{\text{Amount of drug initially taken to prepare the polymersomes}}\times 100$$

### 2.7. In vitro drug release

Paclitaxel-loaded polymersomes (containing 0.1 mg of PTX in 500 μL of solution) or equivalent amount of free PTX were filled in the dialysis bag with a cutoff size of 3.5 kDa and put into 20 mL of phosphate buffer solution at pH 5.0 and pH 7.4 and stirred at 100 rpm. At fixed time interval 10 mL of the buffer solution was withdrawn and replaced with fresh buffer. The drug release was assayed UV spectrophotometrically at the λ_max_ value of 227 nm. The experiments were made triplicate and average values were taken.

### 2.8. Cell culture

Murine glioma cells GL261, human glioma cells U87 (American Type Culture Collection, ATCC) were cultured in DMEM medium containing 10% fetal bovine serum in a humidified incubator at 37 °C with 5% CO_2_. The rat pheochromocytma cell PC-12 cells were maintained in Dulbecco’s modified Eagle’s medium (DMEM) supplemented with 10% (v/v) fetal bovine serum, 10% horse serum, 100 units/ml penicillin and 100 μg/ml streptomycin at 37 °C in a humidified 5% CO_2_ atmosphere.

### 2.9. In vitro cytotoxicity

GL261 cells, U87 cells, or PC-12 grown in 96-well plates at a seeding density of 5 × 10^3^ cells in 200 μL medium per well overnight prior to MTT assay. When reaching to 70%–80% confluency, the cells were exposed to varying concentrations of PTX-containing formulations for 24 h, followed by the addition of 20 μL of MTT solution (5 mg/mL). After incubation for 4 h, the MTT containing medium was aspirated, and 150 μL of dimethyl sulphoxide (DMSO) was immediately added to solubilize MTT formazan produced in viable cells. The absorbance of formazan at 490 nm was measured with a background correction using a Bio-Tek ELX800 ELISA reader. The cell viability was expressed as a percentage of the viable cells in the treated group to the untreated control group.

### 2.10. Apoptotic assay

GL261 cells (1 × 10^5^ cells per well) were seeded in 6-well plates. After 24 h of culture, the cells were incubated with various formulations of PTX (5 μg/mL) in DMEM medium for another 24 h. Apoptotic cancer cells under PTX treatments were detected using an Annexin V-FITC/PI apoptosis detection kit in accordance with the manufacturer’s protocol. Briefly, cancer cells treated with PTX were trypsinized and centrifuged at 1000 rpm for 3 min. The cell pellet was washed with PBS twice and re-suspended in 400 μL binding buffer, and next incubated with 5 μL of annexin V-FITC for 15 min, which was followed by staining with 10 μL propidium iodide (PI) for 5 min in the dark. Then, the samples were analyzed by flow cytometry (FACS-Calibur Instrument, Beckman, USA). Acquired data were analyzed using FlowJo software v10 (FlowJo LLC).

### 2.11. Luciferase assay

Prior to detection, GL261 luciferase-expressing cells (GL261.luc) cells were grown for 24 h in a 96-well black plate at a density of 5 × 10^3^ cells per well. After treated with various PTX groups for another 24 h, luciferase activities of GL261.luc cells were assessed with a substrate d-luciferin using an ELISA reader with luminescence detection (Synergy H1, Bio-Tek, USA).

### 2.12. Cellular uptake study

GL261 cells were seeded in 12-well plates at a density of 1.5 × 10^5^ cells per well, and incubated in 1 mL of DMEM medium containing 10% FBS for 24 h. Subsequently, the culture medium was replaced by fresh DMEM medium containing coumarin-encapsulated polymersomes. After 30 min, 4 h and 24 h incubation, the cells were harvested for flow cytometry quantitative analysis (FACS-Calibur Instrument, Beckman, USA). The data were analyzed with software Flowjo (Version 1.2, Beckman, USA)

### 2.13. Lysotracker staining

GL261 cells were seeded in 8-well chamber slides (Nunc™ Lab-Tek™ II, Thermo Fisher Scientific, USA) at a density of 5 × 10^4^ cells/well. After 24 h culture, the cells were treated with the varying concentrations of fluorescent coumarin-labelled polymersomes. At the exposure time *t* = 30 min, 2 h or 4 h, the cells were pre-stained with LysoTracker Red (final concentration, 1 μM) for 30 min at 37 °C. Subsequently the cells kept in the PBS (10% FBS, v/v) were immediately observed using confocal laser scanning microscopy (FV3000, Olympus, JPN). The images were analyzed with software Image J (NIH, USA).

### 2.14. Detection of ROS levels

Prior to ROS detections, GL261 cells were treated with various formulations at a final concentration of 5 μg/mL of PTX for 24 h. Then the cells were further incubated with DCFH-DA at a final concentration of 10 μM in culture DMEM for 30 min. Intracellular localization of DCF was imaged with a confocal laser scanning microscopy (FV3000, Olympus, JPN), fluorescent intensities were quantified by Image J (NIH, USA).

### 2.15. Immunofluorescent staining

After fixation, cells were rinsed with 10 mM glycine in 0.1% BSA in PBS, and permeabilized with 0.1% Triton X-100 in PBS. Then the cells were subsequentially incubated with primary antibody at 4 °C overnight, and fluorophore-tagged secondary antibody at 37°Cfor 1 h. Filamentous actin was visualized by incubating samples with fluorophore-conjugated phalloidin. Cell nuclei were stained by a DNA probe DAPI.

### 2.16. Western blotting

GL 261 cells were cultured in 6-well plates at a seeding density of 2 × 10^5^ cells per well for 24 h. Next the cells were replaced with nanoformulations containing PTX. After 24 h treatment, the cells were lysed with RIPA buffer on ice for 30 min supplemented with complete protease inhibitor cocktail and 1 mM PMSF. Cell lysates were centrifuged at 15,000 rpm for 15 min at 4 °C. SDS-polyacrylamide gel electrophoresis was carried out with 70 μg of whole-cell lysate from each sample and gels were transferred to PVDF membranes before blocking for 1 h at room temperature. The membranes were incubated with primary antibodies at 4 °C overnight and then hybridized with appropriate HRP-conjugated secondary antibody at room temperature for 2 h. Blots were scanned on gel imaging system (4600, Tanon, China). Images were analyzed with software Image J (NIH, USA). The background of the blots was subtracted by the rolling ball subtraction method with a radius of 50 pixels. Then, the integrated intensity of the bands was measured.

### 2.17. In vivo evaluation

C57BL/6 male mice with 4–5 weeks of age were purchased from the Laboratory Animal Center of Sun Yat-sen University (Guangzhou, China). All the in vivo studies were carried out in accordance with the Guide for Care and Use of Laboratory Animals which were approved by the Institution Animal Care and Use Committee of Sun Yat-sen University. To establish orthotopic glioma murine model, the mice were intracranial injected with 1 × 10^6^ of GL261 cells into ventral striatum. At day 10 post-implantation, various PTX-containing formulations including free PTX, PTX@PS, PTX@LS (Lipusu®) at a PTX dose of 1 mg/kg and a total volume of 5 μL were injected in the same implanted site. After 20 days treatments, all the mice were sacrificed, while brains and other major organs were excised and fixed for one month in neutral sodium salt-buffered formalin. Afterwards, samples were embedded in paraffin wax, and were cut into 10-μm sections. The sections were further dewaxed and rehydrated through xylene and alcohols to be finally washed in running tap water. Hematoxylin and eosin (H&E) staining was used for the histopathological analysis. In addition, the expressions of Ki67, BCL-2, MDR-1 and CD31 in tumor tissues were detected by immunohistochemical staining. The most representative areas of the stained section were selected and marked for analysis. According to the cell staining intensity, the score was grade into 4 levels. 0 point for no positive staining (negative), 1 point for light yellow (weak positive), 2 points for brown yellow (positive) and 3 points for Brown (strong positive). The percentage of positive cells was also rated as 4 levels. 1 point for ≤ 25%, 2 points for 26%–50%, 3 points for 51%–75%, and 4 points for > 75%. The final score is obtained by multiplying the two scores.

### 2.18. Statistics analysis

All the quantitative results were obtained from at least three independent samples. Data were expressed as the mean ± standard deviation (S.D.) and were assessed using Student’s *t*-test (two-tailed). A value of *p* < 0.05 was considered to be statistically significant.

## 3. Results and discussion

### 3.1. Preparation of PTX-loaded polymersomes

**Scheme 1.**
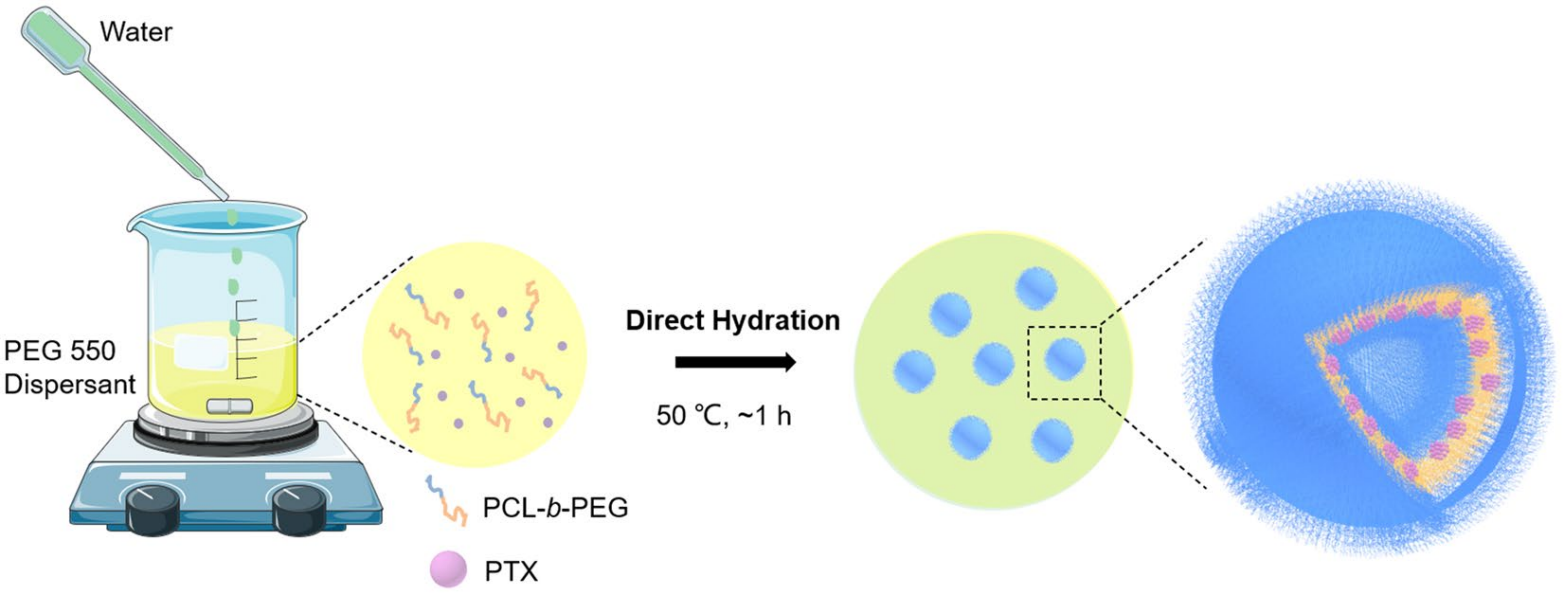
Preparation of PTX-loaded PEG-b-PCL polymersomes.

**Figure 1.**
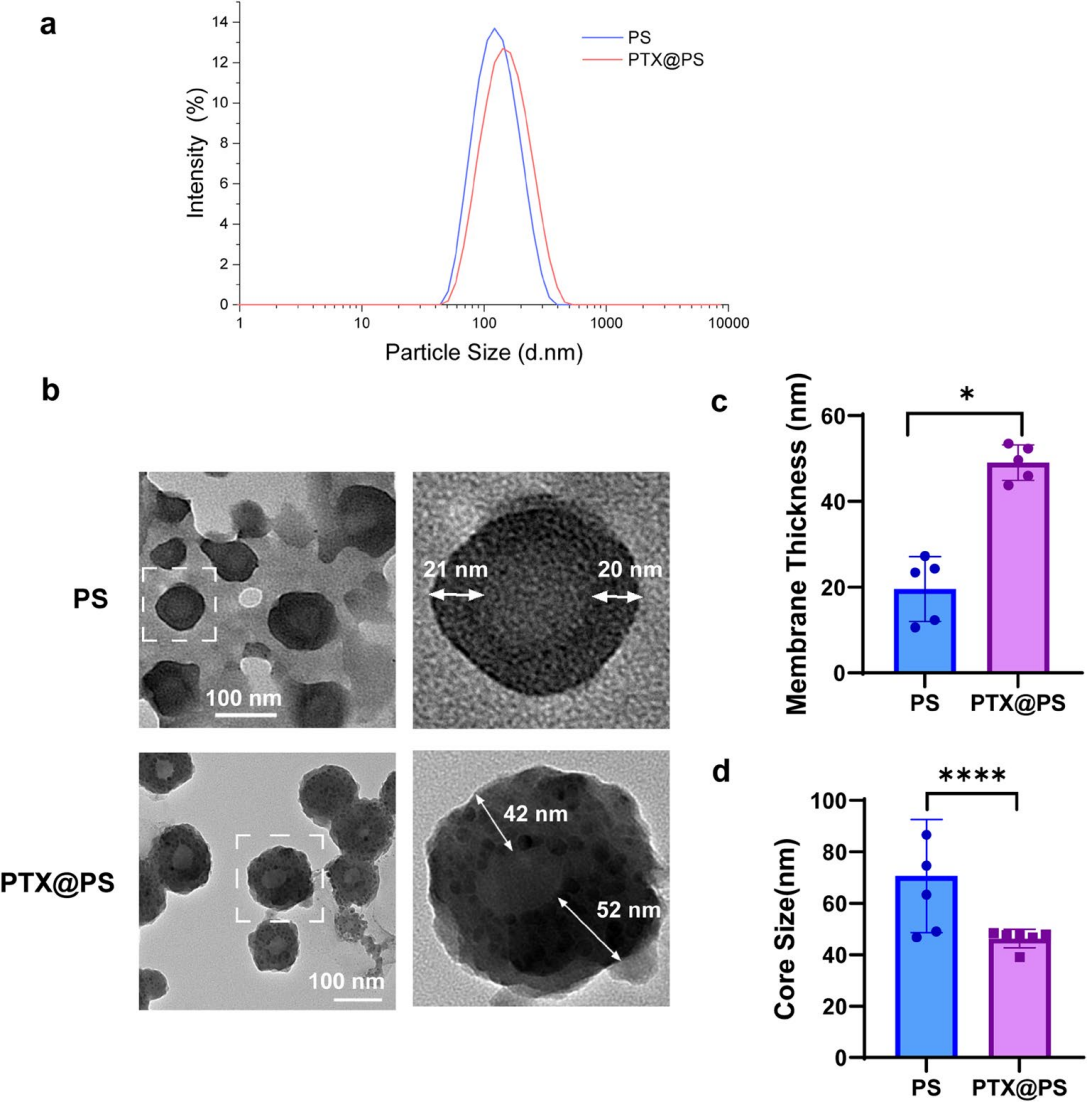
(a) Particle distribution of PEG-*b*-PCL Polymersomes (PS) and PTX-loaded PS (PTX@PS); (b) TEM images of PS and PTX@PS; (c and d) Membrane thickness and core size of PS and PTX@PS. All data were expressed as the mean ± standard deviation (SD.D.). Statistical significances were analyzed using a Student’s’ t-test. * p < 0.05, ****p < 0.0001.

Polymersomes formation is depending on the ratio of hydrophilic and hydrophobic block of polymers. In Sui’s study (Sui et al., 2015), it was found that the polymers with PCL/PEG molecular weight ratios from 2.0 to 5.0 were able to form the polymersomes structure. In view of impact of payload on polymersomes formation, PEG_2k_-*b*- PCL_4.3k_ with a low hydrophobic weight was selected to construct the polymersomes. In this study, PEG*-b*-PCL was synthesized as described previously (Sui et al., 2015). Briefly, the ring-opening polymerization was initialized by the PEG monomethyl ether (MeO-PEO_44_-OH, Mn = 2000) using methanesulfonic acid (MSA) as a catalyst, as illustrated in Scheme S1. The yielded polymer PEG-*b*-PCL was characterized by ^1^H-NMR and GPC. Figure S1 showed ^1^H NMR spectrum of a PEG-*b*-PCL block copolymer and the assignment of the resonances, indicating the polymer had been successfully synthesized. GPC analysis indicated a slightly lower molecular weight of PCL_3.6k_-*b*-PEG_2k_ as 5560 than the expected theoretical value of 6300 (Figure S2). Next, we had applied a direct hydration method to prepare PTX-loaded PEG-*b*-PCL polymersomes (Scheme 1). The obtained polymersomes were further characterized by dynamic light scattering (DLS), showing an average particle size of 118.3 ± 0.97 nm with a narrow PDI value of 0.11 (Figure 1a and Table 1). Moreover, PEG-*b*-PCL polymersomes through direct hydration were vesicle-like polymeric capsules with a membrane thickness of around 20 nm by TEM observations (Figure 1b). Therefore, the results of particle size, size distribution and morphology confirmed that nanosized, vesicles-like shape polymersomes were formed, although PCL/PEG block weight ratio was 1.8 slightly smaller than the predicted values (2.0–5.0).

**Figure 2.**
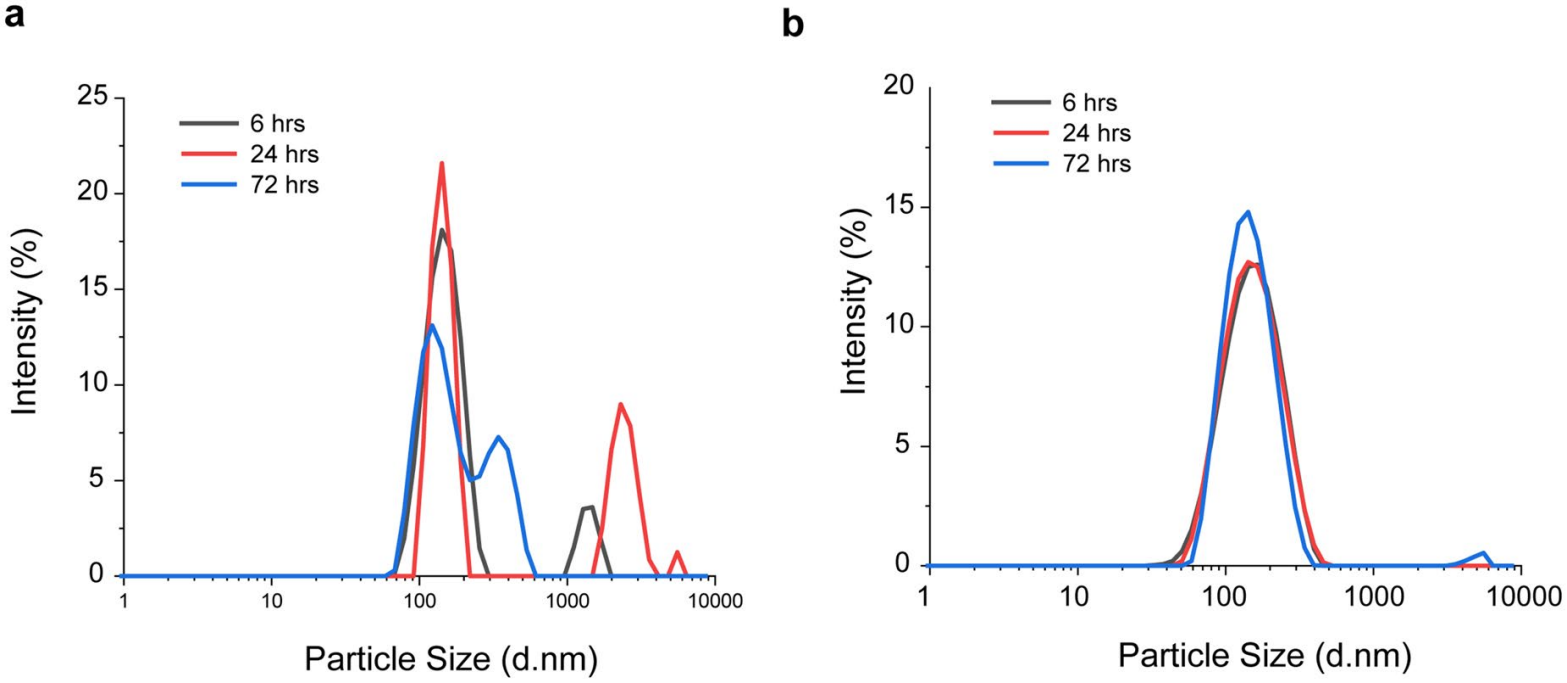
Particle size distribution of PTX loaded in liposomes (Lipusu®) (a) and polymersomes (b) after long-term storage.

**Table 1.** Particle characterization of PS and PTX@PS.

	Size(nm)	PDI	Zeta potential(mV)	LE (%) ^a^	EE (%) ^b^
PS	118.3 ± 0.97	0.11 ± 0.01	−7.74 ± 0.56	/	/
PTX@PS	136.5 ± 0.44	0.16 ± 0.01	−9.23 ± 0.41	2.08 ± 0.14%	39.93 ± 2.43%

LE^a^, Loading Efficiency; EE^b^, Encapsulation Efficiency.

Then we had further tested the drug loading contents and encapsulation efficiency of PEG-*b*-PCL polymersomes, which were two critical factors to evaluate the therapeutic potential of nanoformulations. Although there is no consensus among different studies regarding on drug encapsulation capacity of different polymeric drug carriers (Buzza et al., 2013), PEG-*b*-PCL polymersomes based-drug delivery systems demonstrated a moderate PTX encapsulation content of up to 40% corresponding to a loading efficiency of 2.08% (Table 1). In comparison with the thin-film hydration method, however, using direct hydration method showed a small increase of approx. 10% in PTX encapsulation (Table S1). Currently direct hydration method for polymersomes preparation is in developing, for this reason there is still a need to improve the drug loading based on the full understanding of the drug-loading mechanism in future work. Furthermore, we found that introduction of hydrophobic drug PTX seemed to lightly influence the polymersomes structure and particle dispersity (Figure 1a, b and Table 1). The hydrodynamic size increased from 118.3 nm to 136.5 nm as accompanied by a thicker vesicles membrane around 49.0 ± 4.1 nm and a smaller core after PTX encapsulation (Figure 1(c,d)). Intriguingly, we observed that PTX tended to cluster in the polymeric membrane, representing as a novel form of core-satellite structure. We speculated this drug loading pattern might be contributed by the different hydrophobicity degrees between PTX (log *P* = 7.22) and PCL (log *P* = 4.03). In the process of direct hydration by gradually adding aqueous solution, PTX with high hydrophobicity seems to initially form aggregates, and these aggregates instead of free molecules together with PCL block self-assemble into membrane layers. Although polymersomes are usually obtained by the thin-film hydration method (Lefley et al., 2020), this method, in our hands, produced the polymersomes with a relatively larger diameter of 625.9 ± 48.51 and a wider particle distribution (Figure S3 and Table S1). Moreover, through a thin-film hydration method, there was no structural features of PTX to be identified in TEM images (Figure S4), probably because PTX was distributed throughout the polymersomes membrane. With respect to particle size and homogeneity related with stability issues, thereby, direct hydration method seemed more competent to construct polymersomes to deliver drug compared to the conventional thin-film hydration method. Additionally, no significant differences in surface charges were noted between the two formulations with zeta potential values around −7.0 mV, indicating there was no electronic interaction between drug and polymers.

**Figure 3.**
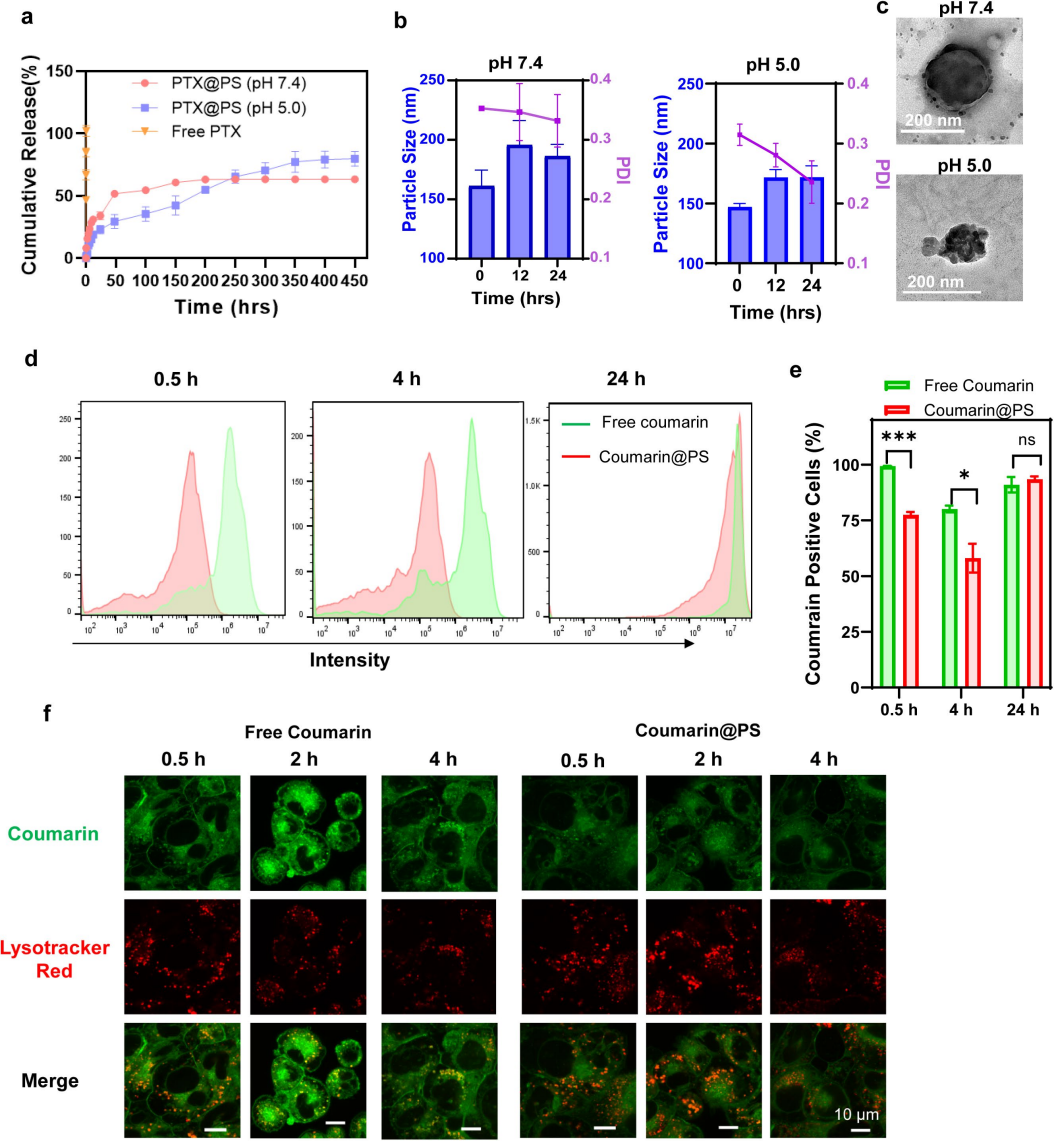
(a) In vitro release kinetics of PTX@PS and PTX alone at pH 7.4 or pH 5.0; (b-c) Particle size and morphology of PTX@PS at varying pH with time increasing; (d-e) FACS analysis and relative quantification of free coumarin and coumarin loaded in the polymersomes (Coumarin@PS) at a coumarin concentration of 1 μg/mL in GL 261 cells; (f) CLSM images of GL261 cells treated with free coumarin or coumarin-labelled polymersomes (green) for 30 min, 2 h and 4 h. The cells were stained with lysotracker red. Scale bar was 10 μm. All data were expressed as mean ± SD. Statistical comparisons were conducted using the student’s *t*-test, *p < 0.05, ***p < 0.001, ****p < 0.0001, ns = not significant.

**Figure 4.**
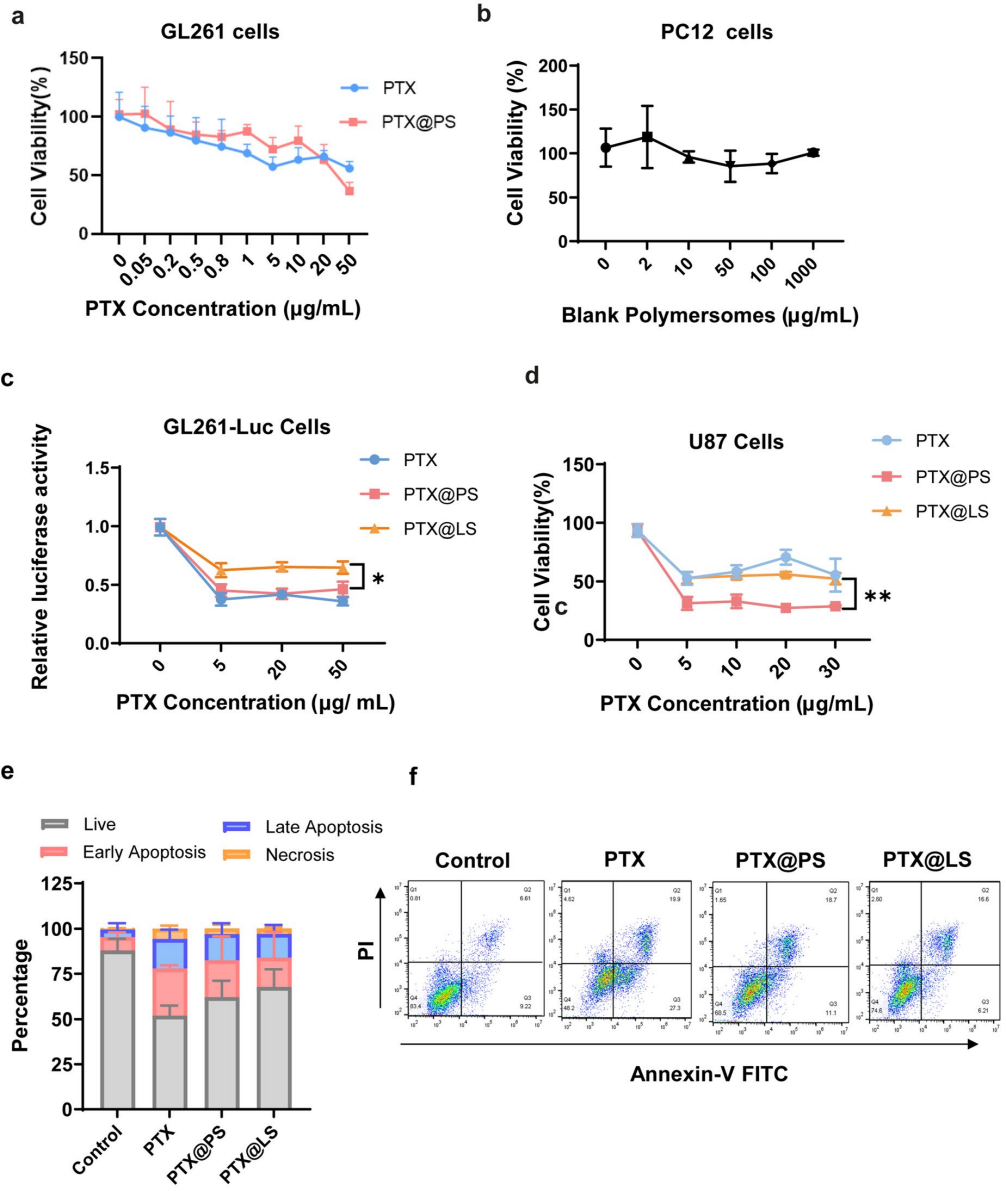
(a) Cytotoxicity of PTX or PTX loaded in poymersomes (PTX@PS) were evaluated in GL 261 cells by MTT assay; (b) Cell viability of blank polymersomes in PC 12 cells. (c) and (d), Cytotoxic effects of PTX-containing various formulations with a range of concentrations (from 5 to 50 µg/mL) in GL261-Luc cells and U87 cells. (e) and (f), Apoptotic assay of PTX-containing various formulations at a PTX concentration of 5 µg/mL in GL261 cells. All data were expressed as mean ± SD, * *p* < 0.05, ** *p* < 0.01 versus PTX@ LS (Lipusu^®^).

Moreover, we tested the physical stability of polymersomes and liposomes after the long-term storage by measuring average size as well as their size distribution, since these parameters could be used to monitor the functions of drug nanocarrier. After suspended in PBS buffer, freeze-dried pellets PTX@LS (Lipusu^®^) showed a particle size of 583.5 ± 155.1 nm with a broader particle distribution (PDI = 0.79). Moreover, there is a higher population (around 31%) of micron aggregations compared with PTX@PS (Figure 2). Although lyophilization is widely utilized to prolong shelf life of liposomes by reducing lipid oxidation, the instability accompanied by rehydration seemed to be inevitable and were commonly found in many liposomal formulations (Ghanbarzadeh et al., 2013). In contrast, PTX@PS could be rapidly produced in 1 h under mild conditions, and specifically exhibited high reproducibility and stability. Additionally, PTX highly dispersed throughout the polymeric matrix as a core- satellite might also contribute to the colloidal stability. Likewise, immobilization of gold nanoparticles (Wu et al., 2017), silver nanoparticles (Zhang et al., 2017), iron nanoparticles (Sun et al., 2016), silicon nanoparticles (Ge et al., 2008; Herrmann et al., 2017), and DNA (Tang et al., 2017) in the coating layers enable to stabilize the core nanoparticles. Despite these nano-systems fabricated by different materials, most are metals, there is consensus that the core–satellite superstructure maintains the spherical shape and shows an improved stability. Therefore, this ready-to-use preparation of polymersomes appeared to be a promising alternative to liposomes as drug carriers.

### 3.2. PTX-loaded polymersomes showed sustained drug release

Controlled and sustained drug release is a desirable feature for polymeric drug carriers. The pH values, i.e. physiological pH 7.4 and intracellular endolysosomal pH (4.0–5.5) could significantly influence the stability and drug release. We have examined the drug release profiles of PTX-loaded polymersomes under different pH conditions at varying time points. In Figure 3(a), a rapid release was showed in the free PTX solution with nearly 100% of drug release in 1 hour. On the contrary, PTX@PS showed a cumulative drug release of 51.72% at pH 7.4 in the initial 48 h, and continuously released additional 10% of total drugs up to 200 hours. Strikingly, upon exposure to acidic microenviroments (pH 5.0), the formulation extended the release up to 79.79% of drug within 450 hours (approx. 19 days). Although both release profiles at pH 7.4 and those at pH 5.0 were best fitted to the Higuchi Model suggesting a mechanism of drug release through wetting and diffusion (Figure S5), we inferred this acidity-induced sustained release profiles were possibly contributed by the slow hydrolysis rates of PEG*-b-*PCL polymers under pH 5.0, since breakdown of ester bonds was reduced by decreasing pHs (Rydholm et al., 2007; White et al., 2021). Additionally, the pH-dependent release profiles could be also elucidated by an increase in polymersome size and polydispersity index at pH 7.4 as compared to those at pH 5.0 (Figure 3b). Therefore, in combination with TEM observations (Figure 3c), our results demonstrated that polymersomes at pH 7.4 within 50 h were prone to enlarge, and drug pellets were dissociated from polymeric membrane and scattered outside the polymerdsomes, whereas polymersomes morphology at pH 5.0 seemed to be destructed but without a great amount of drug leakage. Additionally, we found that there was no complete drug release with PTX@PS at any pH, probably because PCL had a long degradation time of three to four years (Arakawa & DeForest, 2017; Brigham et al., 2021). Thereby, we assumed that the core-satellite superstructure might prolong the drug release PTX@PS. PTX self-assembled into micellar-like aggregates and were further embedded into the polymersomes membrane, therefore, PTX would have to dissociate from aggregates and permeate through the polymeric membrane, instead of a single one before leaking into the outside environment. A similar phenomenon was also observed in previous study (Marguet et al., 2012) that doxorubicin (Dox) was encapsulated in the multiple polymersomes, which consisted of inter nanovesicles and outer giant polymersomes, which demonstrated a controlled permeability and a decreased drug release rate.

**Figure 5.**
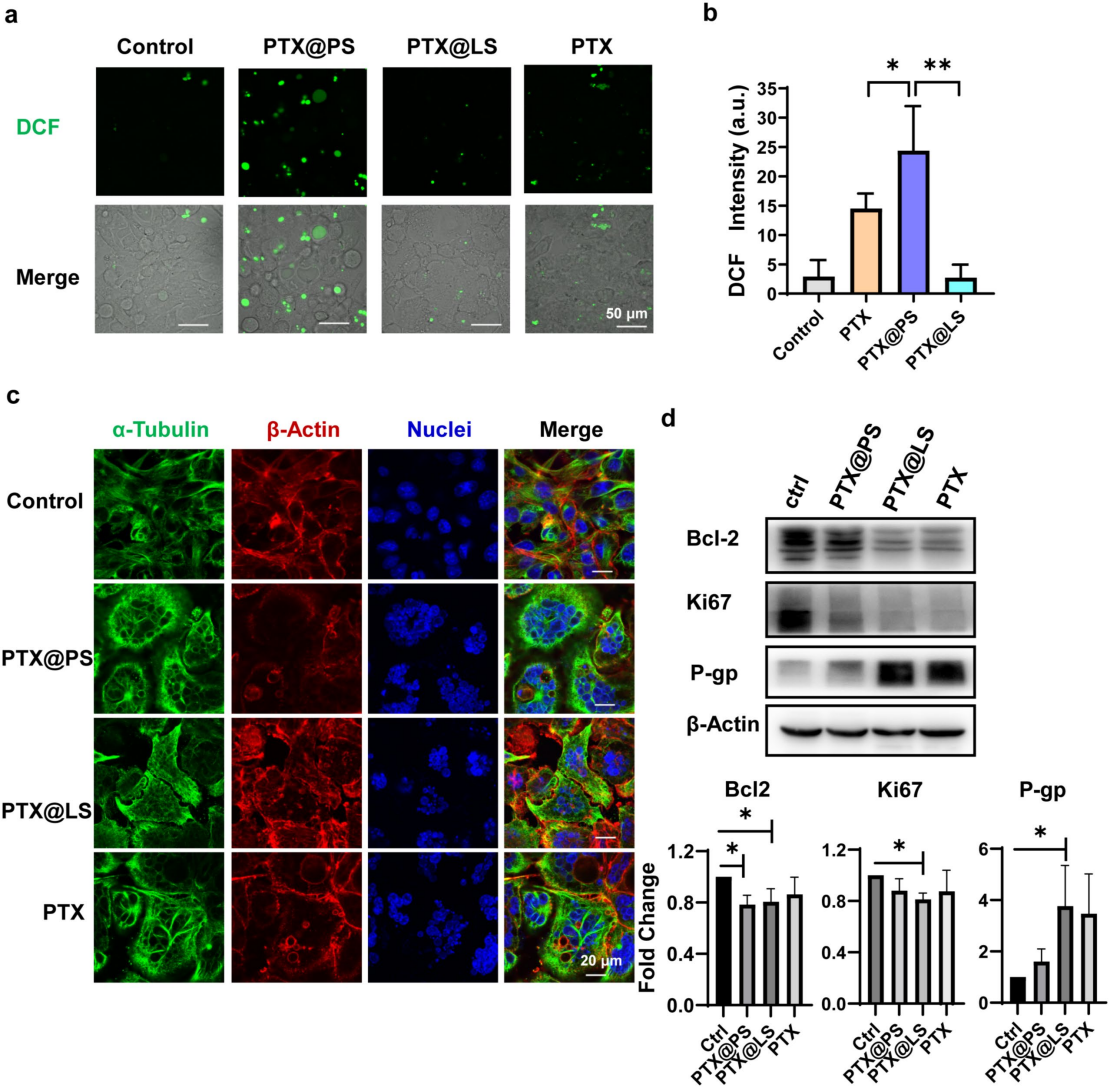
(a) and (b) Detection of ROS production using DCFH-DA assay, scale bar was 50 μm; (c) Confocal laser scanning images of microtubules stained with α-tubulin (green), endogenous actin filaments using phalloidin (red) and DAPI (blue), scale bar was 20 μm; (d) Western blotting of Blc2, Ki67 and P-gp. The target protein expressions were quantified and normalized by β-Actin. All data were represented as mean ± SD, * *p* < 0.05 determined by *t*-test.

Next, fluorescent dye coumarin-labeled polymersomes were used to investigate the cellular uptake mechanism and intracellular fate. Internalization of coumarin loaded polymersomes (coumarin@PS) was evaluated with FACS after 30 min, 4 h and 24 h (Figure 3(d,e)). Although the fluorescent positive cells treated with coumarin@PS were significant less than those with free dye at early time points (30 min and 4 h), with increasing time it showed comparable cellular accumulation after 24 h. This observation was in accord with previous findings (Zhang et al., 2018), probably because highly hydrophobic fluorescent dye was prone to rapidly enter GL261 cells through passive diffusion, but coumarin@PS were slowly taken up into cells through endocytosis. Furthermore, cellular release behavior of coumarin@PS at early times was monitored by CLSM observations (Figure 3f). It revealed that free dyes were mainly localized in the cytosol, and less in the endolysosomes which were stained with lysotracker red, indicating a cooccurrence of multiple internalization pathways. Similarly, the cells treated with courmarin@PS were also found that a number of coumarin distributed throughout the cytosol within 30 min, suggesting that the polymersomes were capable of endosomal escape for cargo delivery to cellular target, i.e. microtubules in this case. It is unlikely that coumarin was released from polymersomes in the extracellular space and diffused into the cell, since there was no burst release at pH 7.4 in the first 30 min. Endocytic processes of PTX@PS seemed to contribute to overcome drug resistance, since passively transferred drugs across cell membrane were easy to activate drug transporters to export drug out of the cells (Gottesman & Pastan, 2015). Therefore, the time-dependent endocytosis behavior and controlled drug release in cells could provide guidance for further anticancer therapy.

### 3.3. PTX-loaded polymersomes exerted anti-tumor effects on glioma cells

To evaluate the pharmacological activity of PTX-loaded polymersomes, in vitro cytotoxicity tests against the growth of murine glioma cells GL261 cells and human glioma cells U87 were conducted. After treated with PTX@PS and PTX alone at the PTX concentrations ranging from 0 to 50 μg/mL for 24 h, cell viability of GL261 cells was measured by MTT assay. IC50 values of PTX@PS and free PTX were calculated as 23.07 and 26.48 μg/mL, respectively (Figure 4a). Correlating with cytotoxicity, we next examined the luminescence intensities of luciferase-expressing GL261 cells exposed to various formulations. The intensity of PTX@PS was significantly lower than liposomal PTX, and was similar to that of free PTX (Figure 4c). In contrast to GL261 cells, the cell proliferation of U87 cells was remarkably decreased after addition of PTX@PS compared with PTX alone or PTX@LS, indicating polymersomes-based formulation augments the sensitivity of U87 cells to PTX. Consistent with the results in cytotoxicity evaluation, PTX@PS at a PTX concentration of 5 μg/mL caused moderate apoptotic rate of GL261 cells (37.95% of total cell numbers) compared with commercial liposomal formulation (32.35%), but was still less efficient than free drug (Figure 4(e,f)). Due to high cell permeability, free PTX could largely accumulate into the cells, therefore these cells rapidly underwent the early process of apoptosis. Moreover, there was no significant toxicity of blank polymersomes observed in PC 12 cells (Figure 4(b)). Although PTX@PS did not exhibit superb cell killing ability compared with free drug in vitro, polymersomes were able to improve the bioavailability and solubility. Consistently, many researches had shown that liposomal formulations are less toxic than drug alone but improved pharmacological profiles of the drug (Zylberberg & Matosevic, 2016; Beltrán-Gracia et al., 2019). Therefore, our results demonstrated chemotherapeutic potential of PTX@PS associated with an increase in apoptotic rate and a decrease in proliferative index compared with PTX@LS (Lipusu^®^).

Nanomaterials induced oxidative stress could exert either pro-tumor or anti-tumor effects (Aboelella et al., 2021). Cellular ROS production by PTX-treated glioma cells was detected using DCFH-DA assay. In contrast to free PTX or PTX@LS, total intracellular ROS levels were significantly elevated in the cells incubated with PTX@PS (Figure 5(a,b)). Evidently, PTX triggered-ROS generation was able to synergize the cytotoxicity of PTX. Moreover, disruption of microtubulin assembly dynamics had been considered as a main cause of PTX as an anti-mitotic drug. As shown in Figure 5(c), we examined the tubulins morphology of cells treated with different PTX-containing formulations in an immunofluorescence assay. More tubulin bundles as the remarkable signals of microtubule polymer stabilization were observed in the cells added with PTX@PS and PTX, whereas PTX@LS (Lipusu^®^) treated or untreated cells displayed radial arrays of interphase microbubules. Furthermore, to assess the tumorgenicity of glioma cells treated with PTX-based formulations, we detected the expression levels of anti-apoptotic proteins Bcl2 and cell proliferation marker Ki67 by western blotting. It demonstrated that different types of PTX formulations had varying degrees of impairment on protumor proteins Bcl2 and Ki67 expression (Figure 5d). As we mentioned above, a large amount of free drug would activate drug transporters which was normally involved in multidrug resistance, that is one of the main reasons underlying failure of chemotherapy in cancer patients (Alfarouk et al., 2015). Intriguingly, PTX@PS could completely downregulate the drug transporter P-gp expression induced by anti-cancer drug, which seemed to be a novel therapeutic approach to overcome the drug resistance.

### 3.4. PTX-loaded polymersomes inhibited tumor growth in orthotopic glioma murine models

**Figure 6.**
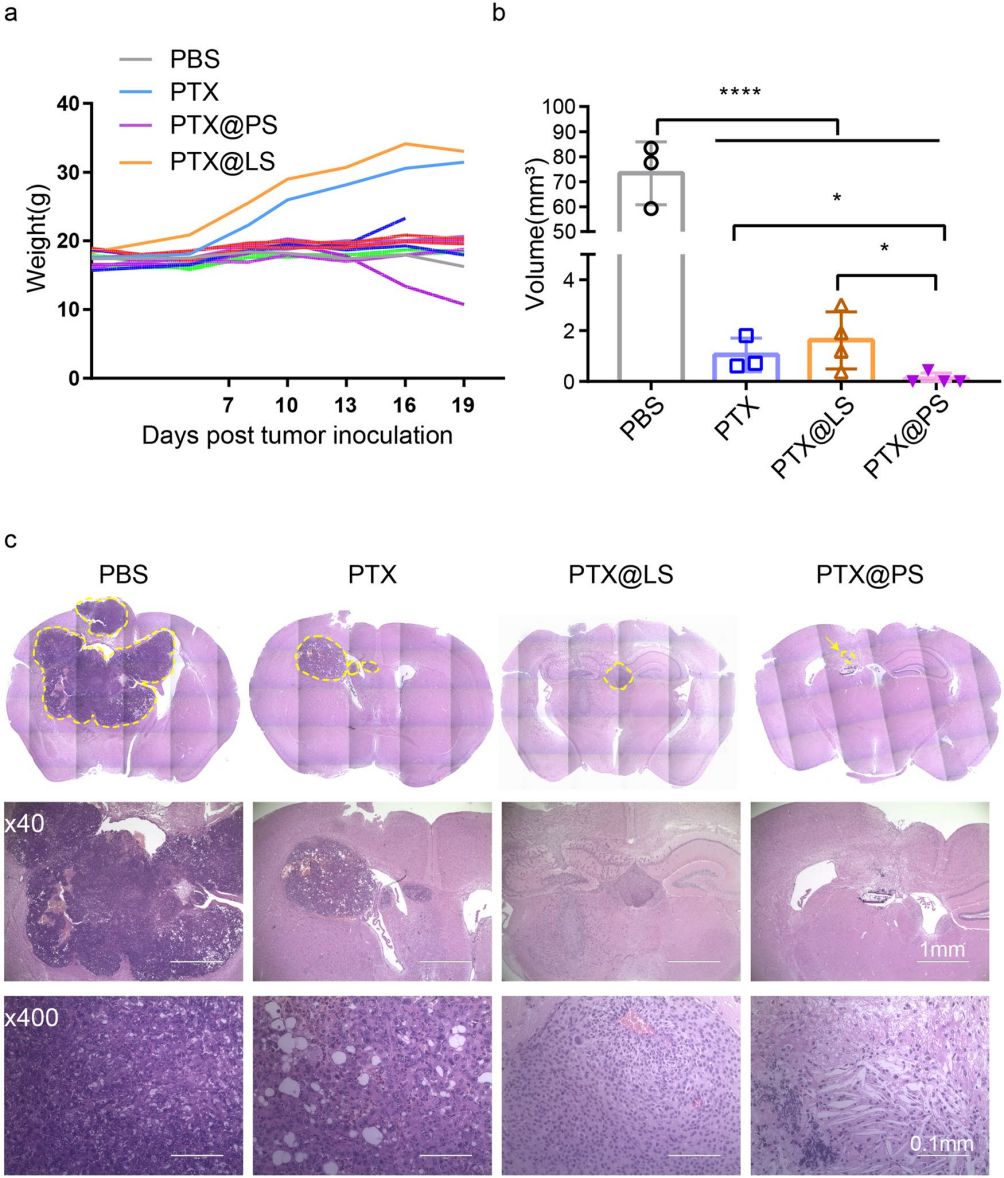
(a) Monitoring of body weights of glioma bearing mice aftr treatments; (b) Tumor volumes were quantified by using a formula: V = (W^2^× L)/2, where V is tumor volume, W and L is width and length of the greatest tumor area in H&E stained slides; (c) Representative images of tumor areas are outlined by yellow dashed lines in H&E stained brain sections. All data were represented as mean ± SD, and asterisks indicate statistical significance by *T* test (*n* = 4, *p < 0.05, ****p < 0.0001).

Surgical resection still remains the first clinical intervention for glioma patients, but often fails to fully remove the infiltrative tumor satellites leading to a relapse. In addition, drug delivery through intravenous routes is required to overcome the blood-brain barrier. Therefore, intracavity implantation appears an effective drug delivery approach. Based on a sustained and controlled release profile, we proposed PTX@PS at the site of glioma resection cavity would eliminate residual tumor cells that infiltrated the brain parenchyma. We have established orthotopic glioma models though intracranial injection of GL261 cells in C57BL/6 mouse. After 10 days of tumor inoculation, various PTX-containing formulations at a PTX dose of 1 mg/kg were in situ injected into the site of tumor implantation. Animals were able to tolerate the surgical procedure, as demonstrated by negligible body loss without mortality (Figure 6a). After 19 days treatments of PTX formulations, the mice were sacrificed, and the brains were excised to further evaluate the tumor size and shape, prognostic assessments and pattern of vascularization. In the PBS buffer treated group, histological analysis showed tumor developed in the area of the residual cavity with a mean volume of 73.39 ± 12.62 mm^3^ (Figure 6(b, c)). These tumors appeared an irregular shape and margins with many tumor cells invading and islets of cells inside the normal brain parenchyma. By contrast, PTX treatments including PTX solution, PTX@LS and PTX@PS significantly inhibited above 97% of tumor growth. In these tumors, it showed a small tumor mass with a well-delineated margins and very little sign of local infiltration. Strikingly, PTX@PS showed the highest effects to eradicate the tumor and no visible tumors were detected in 50% of PTX@PS treated mice.

**Figure 7.**
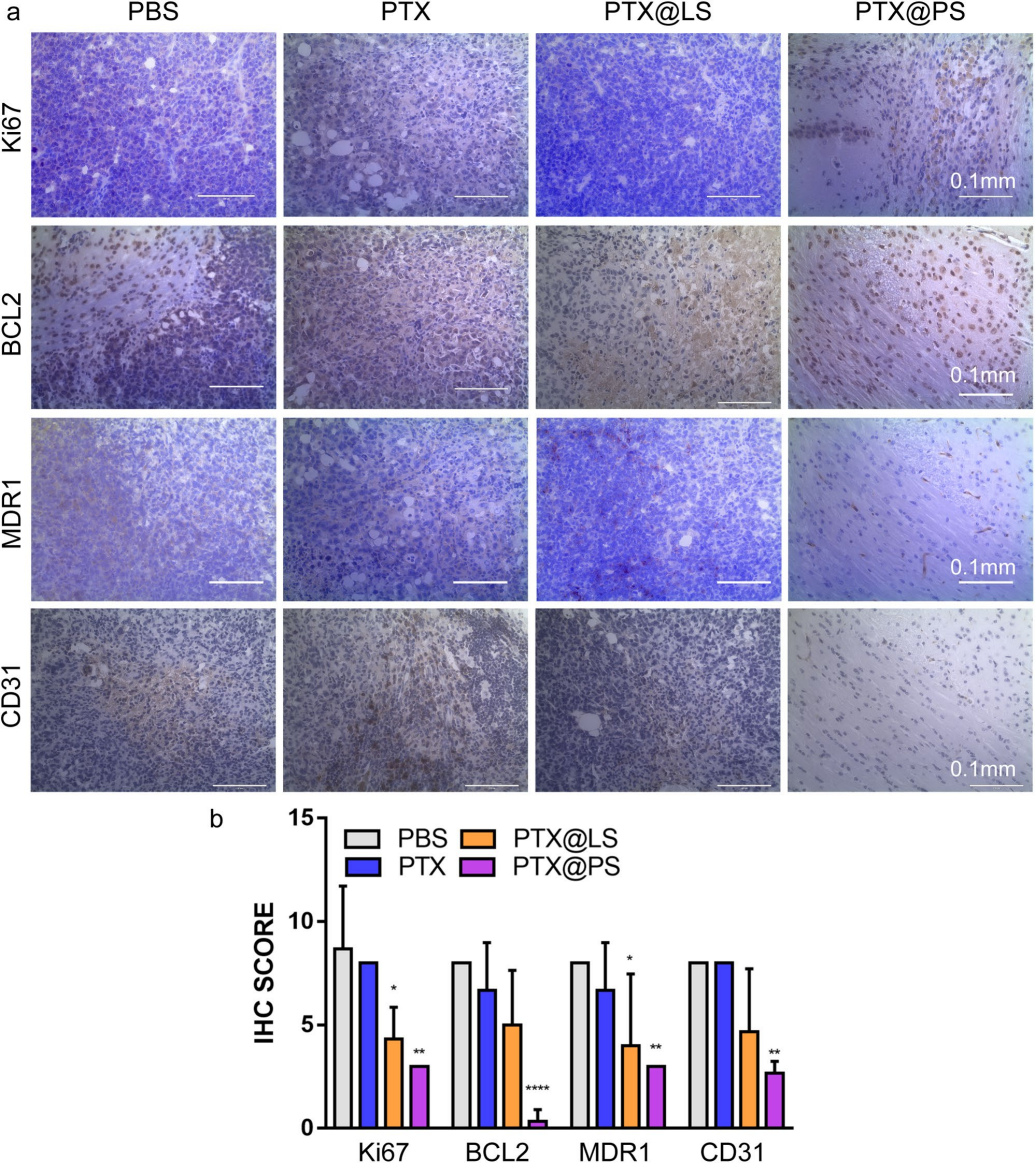
(a) Immunohistochemistry staining with specific antibody against Ki67, Bcl2, MDR1(P-gp) and CD31 in brain glioma derived from animals subjected to various treatments. (b) Immunohistochemical quantification for Ki67, BCL2, MDR1 and CD31 in tumor areas. All data were expressed as mean ± SD, and asterisks indicated statistical significance by one-way ANOVA compared with PBS group (*n* = 4, *p < 0.05, **p < 0.01, **** *p* < 0.0001).

Additionally, we also assessed the expressions of anti-apoptotic marker Bcl2 and proliferative marker Ki67 in order to predict the recurrence (Figure 7a). Bcl2 correlated with Ki67 were strongly expressed in control (PBS injected) group, suggesting a high risk of relapse. In comparison to irregular cellular morphology and looser cell arrangements observed in brain sections from free PTX and PTX@LS injected groups, few Bcl-2 and Ki67-positive tumor cells were identified in PTX@PS treated brains. In line with in vitro results, we found that both PTX@PS and PTX@LS could not induce the upregulation of MDR1/P-gp expressions. Moreover, the brain slices were also further stained with anti-CD31 antibodies to determine the microvessel patterns. In the PTX@PS treated group, vessels were small, composed by unilayer of endothelial cells, and formed a well-defined and regular vascular network. On the contrary, PTX, PTX@LS treated or untreated brain tumors had a heterogeneity of microvessel distribution with irregular shapes and formed a complex network with many telangiectatic vessels and some glomerular structures. These above observations were further confirmed by quantitative scoring that PTX@PS markedly decreased the expression levels of Ki67, Bcl2, MDR1 and CD31 compared to other treatments, implying a lower rate of tumor recurrence in mice (Figure 7b). In addition, histological analysis of the organs including liver, kidney, spleen and lung did not reveal any signs of inflammation or toxicity in PTX@PS treated groups (Figure S6 and S7). Together, the results confirmed that PTX@PS remarkably suppressed glioma growth with a considerable low risk of recurrence.

## 4. Conclusions

In conclusion, this study demonstrated a modified direct hydration method to load anti-cancer drug PTX into PEG-*b*-PCL polymersomes through a self-assembly process without using any organic solvents or surfactant. In comparison to commercial liposomal formulation Lipusu^®^ (PTX@LS), PTX@PS was more stable and toxic on glioma cells through in vitro and in vivo evaluations, which was probably induced by multiple mechanisms including oxidative stress and tubulin polymerization dysfunction. Notably, intracranial treatments of PTX@PS showed above 99% of glioma growth suppression in vivo, since this ready-to-use formulation considerably improved the sustained release of PTX without occurrence of multidrug resistance. Hence, our study provided a simple method for preparing anti-cancer drug encapsulated polymersomes, which represented a promising candidate to replace liposomes as therapeutic drug delivery carriers.

## Supplementary Material

Supplemental Material

## Supplementary materials

Scheme S1: Synthetic scheme of PEG-*b*-PCL Figure S1: 1H-NMR spectrum of PEG-*b*-PCL; Figure S2: GPC elution curve of PEG-*b*-PCL; Figures S3, S4 and Table S1: Particle Characterizations of PTX-loaded polymersomes prepared by a thin-film hydration method; Figure S5: Higuchi model of PTX@PS release profiles at different pH; Figures S6 and S7: Histological analysis of major organs.
