# Supplementary material for: A facile method for anti-cancer drug encapsulation into polymersomes with a core-satellite structure
Source: Drug Deliv. 2022 Jul 29;29(1):2414–27. doi: 10.1080/10717544.2022.2103209 (PMC9341360; doi:10.1080/10717544.2022.2103209)
Supplement: Supplemental Material [file IDRD_A_2103209_SM3669.docx]

**Scheme S1**. Synthetic scheme of PEG-*b*-PCL.


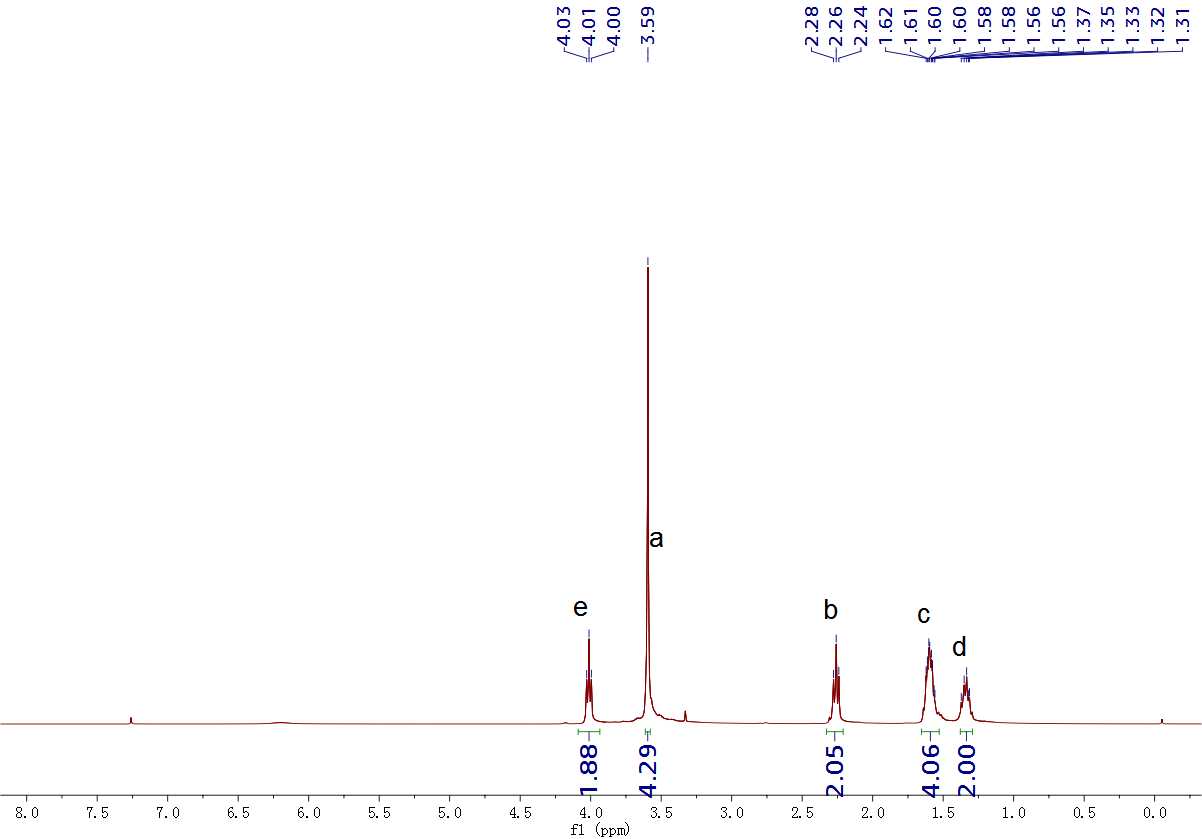


**Figure S1**. ^1^H-NMR spectrum of PEG-*b*-PCL


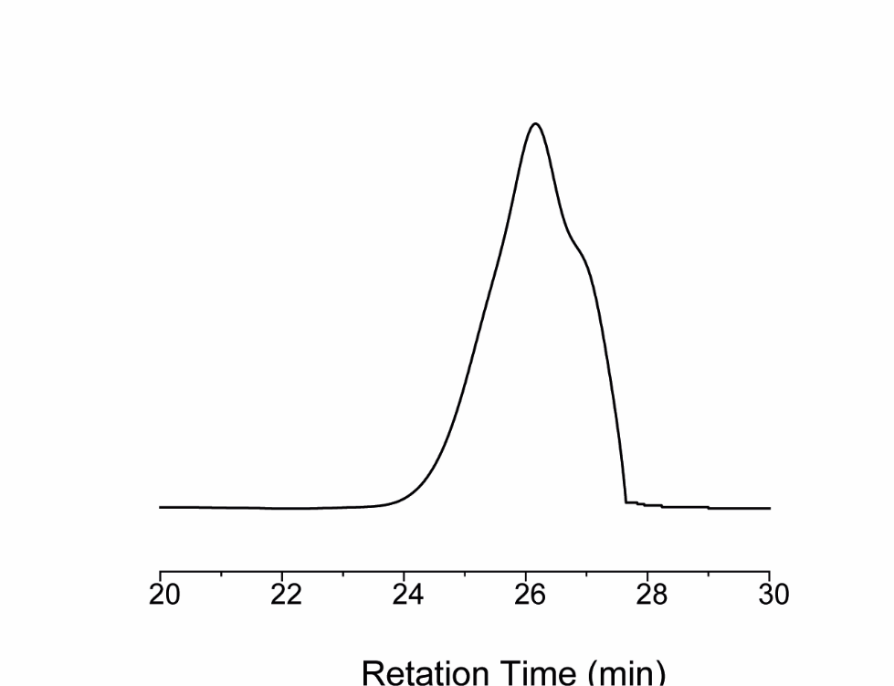


**Figure S2.** GPC elution curve of PCL*-b*-PEG.

Batch1

Batch2

**Figure S3**. Particle distribution of PTX-loaded polymersomes prepared from two different batches by a thin-film hydration method.


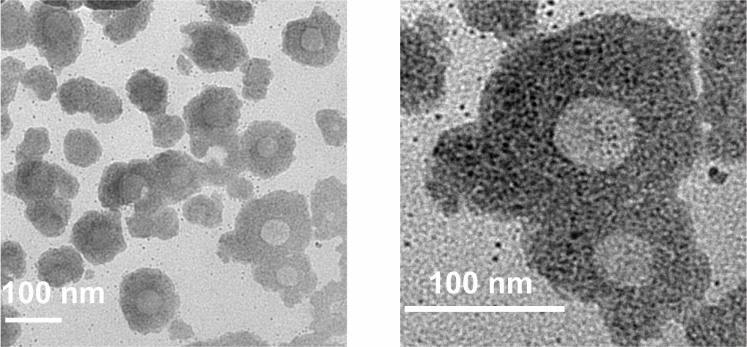


**Figure S4.** TEM images of PTX-loaded polymersomes prepared by a thin-film hydration method.

**B**

Q=3.85t^1/2^+11.94

R^2^=0.90311

**A**

Q=3.87t^1/2^+1.16

R^2^=0.98772

**Figure S5**. Higuchi model of the cumulative percentage of PTX released from PTX@PS at different

pH conditions. A, pH 7.4; B, pH 5.0.


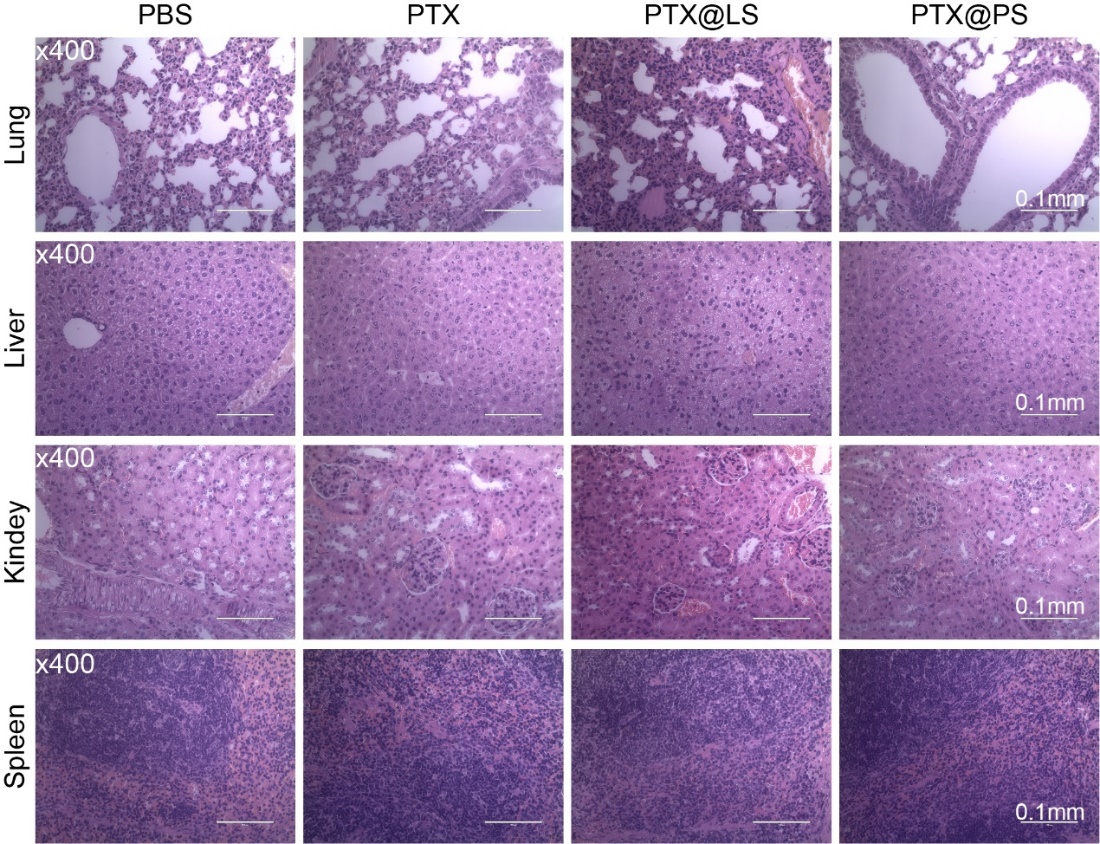


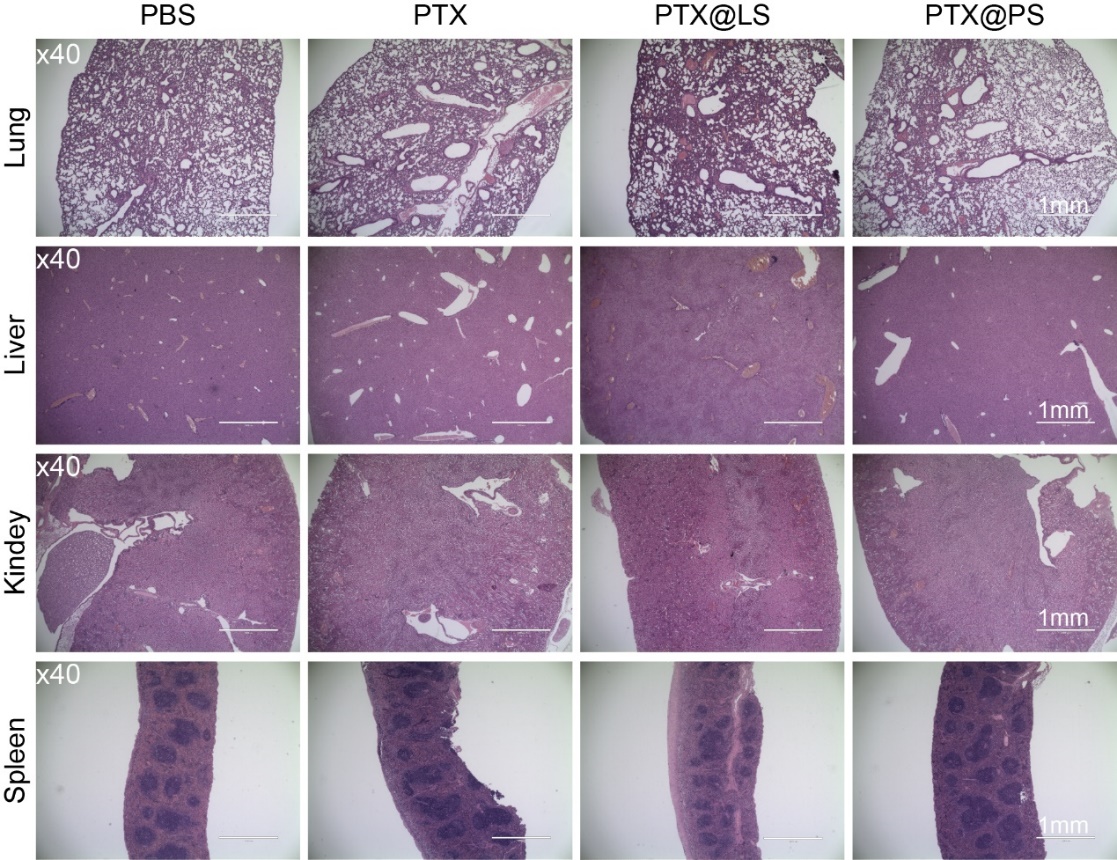


**Figure S6**. Histological analysis of major organs at × 40 magnification.

**Figure S7.** Histological analysis of major organs at × 400 magnification.

**Table S1. Particle characterization of PTX@PS prepared by thin-film hydration method**

|  | Size(nm) | PDI | Zeta potential(mV) | LE (%) ^a^ | EE (%) ^b^ |
| --- | --- | --- | --- | --- | --- |
| PTX@PS | 625.9 ± 48.51 | 0.819 ± 0.313 | -1.44 ± 0.34 | 1.95 ± 0.31% | 28.82 ± 4.63% |

LE^a^, Loading Efficiency; EE^b^, Encapsulation Efficiency.
